# Percutaneous mesh-container-plasty versus percutaneous kyphoplasty for the treatment of Kümmell's disease: a retrospective cohort study

**DOI:** 10.1186/s13018-023-03753-1

**Published:** 2023-03-30

**Authors:** Yimin Li, Yunfan Qian, Guangjie Shen, Chengxuan Tang, Xiqiang Zhong, Shaoqi He

**Affiliations:** Department of Orthopaedic Surgery, Third Affiliated Hospital of Wenzhou Medical University, 108 WanSong Road, Ruian, Wenzhou, Zhejiang China

**Keywords:** Percutaneous kyphoplasty, Percutaneous mesh-container-plasty, Kümmell's disease

## Abstract

**Background:**

Both percutaneous kyphoplasty (PKP) and percutaneous mesh-container-plasty (PMCP) were important procedures for the treatment of Kümmell's disease. This study aimed to compare the clinical and radiological results of PKP and PMCP for the treatment of Kümmell's disease.

**Methods:**

This study included patients with Kümmell's disease treated at our center between January 2016 and December 2019. A total of 256 patients were divided into two groups according to the surgical treatment they received. Clinical, radiological, epidemiological, and surgical data were compared between the two groups. Cement leakage, height restoration, deformity correction, and distribution were evaluated. The visual analog scale (VAS), Oswestry Disability Index (ODI), and short-form 36 health survey domains “role-physical” (SF-36 rp) and “bodily pain” (SF-36 bp) were calculated preoperatively, immediately after surgery, and 1-year postoperatively.

**Results:**

The VAS and ODI scores improved in the PKP [preoperative: 6 (6–7), 68.75 ± 6.64; postoperative: 2 (2–3), 23.25 ± 3.50, respectively] (*p* < 0.05) and the PMCP [preoperative: 6 (5–7), 67.70 ± 6.50; postoperative: 2 (2–2), 22.24 ± 3.55, respectively] groups (*p* < 0.05). There were significant differences between the two groups. The mean cost in the PKP group was lower than that in the PMCP group (3697 ± 461 vs. 5255 ± 262 USD, *p* < 0.05). The cement distribution in the PMCP group was significantly higher than that in the PKP group (41.81 ± 8.82% vs. 33.65 ± 9.24%, *p* < 0.001). Cement leakage was lower in the PMCP group (23/134) than in the PKP group (35/122) (*p* < 0.05). The anterior vertebral body height ratio (AVBHr) and Cobb’s angle improved in the PKP (preoperative: 70.85 ± 16.62% and 17.29 ± 9.78°; postoperative: 80.28 ± 13.02% and 13.05 ± 8.40°, respectively) and PMCP (preoperative: 70.96 ± 18.01% and 17.01 ± 10.53°; postoperative: 84.81 ± 12.96% and 10.76 ± 9.23°, respectively) groups (*p* < 0.05). There were significant differences in vertebral body height recovery and segmental kyphosis improvement between the two groups.

**Conclusions:**

PMCP had advantages over PKP in terms of pain relief and functional recovery for the treatment of Kümmell's disease. Moreover, PMCP is more effective than PKP in preventing cement leakage, increasing cement distribution, and improving vertebral height and segmental kyphosis, despite its higher cost.

## Background

Kümmell's disease is a serious complication of osteoporotic compression fractures. It occurs because the vacuum created by the fracture does not heal on its own. The repair mechanism of the diseased vertebra enters a vicious cycle with progressive vertebral collapse, intravertebral pseudarthrosis, kyphosis, and in severe cases, secondary spinal stenosis with neurological symptoms. Therefore, it is also clinically known as non-union of osteoporotic vertebral fractures [1, 2].

Kümmell's disease is divided into three stages according to imaging manifestations [3]: Stage I, vertebral height loss of < 20% without adjacent degenerative disc disease; stage II, vertebral height loss of > 20% with adjacent degenerative disc disease; and type III, intravertebral obstruction and nerve compression, with symptoms of nerve damage when the compression is severe. Conservative treatment for Kümmell’s disease is ineffective; moreover, delayed management may lead to increased spinal deformity, nerve damage, and even paralysis [4–6]. For Kümmell’s disease stages I–II without segmental instability, percutaneous vertebroplasty (PVP) or percutaneous kyphoplasty (PKP) is usually performed [7]. For Kümmell’s disease stage III without neurological impairment, PKP is recommended [8].

Patients with Kümmell's disease have a high rate of cement leakage after PVP and PKP because of vertebral wall insufficiency, especially the rupture of the posterior vertebral wall. Other complications, such as the loss of restored height and kyphotic alignment after balloon deflation and before cement injection, have also been reported [9, 10]. Consequently, a mesh container was developed to reduce kyphotic angles, restore height, and prevent cement leakage [11]. Studies that have compared percutaneous mesh-container-plasty (PMCP) and PKP for the treatment of Kümmell's disease, especially with large sample sizes, are limited [12]. Thus, we aimed to compare the clinical efficacy and safety of PMCP and PKP for the treatment of Kümmell's.

## Methods

### Study design

This study was approved by the ethics committee of the Third Affiliated Hospital of Wenzhou Medical University (YJ2022057).

The inclusion criteria were as follows: (1) age ≥ 60 years; (2) single vertebral involvement without neurological symptoms; (3) primary osteoporosis with bone mineral density T-value ≤ -2.5; (4) magnetic resonance imaging (MRI) or computed tomography (CT) revealed vertebral osteonecrosis with intravertebral vacuum cleft signs [13, 14].

The exclusion criteria were as follows: (1) inability to tolerate surgery; (2) vertebral destruction caused by tumors, spinal infections, tuberculosis, or brucellosis; (3) neurological impairment requiring decompression; and (4) coagulation dysfunction.

Between January 2016 and December 2019, 256 patients with Kümmell’s disease without neurological deficits were included in this study according to the inclusion and exclusion criteria. Of these 256 patients, 122 underwent PKP and 134 underwent PMCP. Before surgery, all the patients were informed of the differences between PKP and PMCP, and the surgical methods were selected according to their preferences.

Preoperatively, the patients underwent electrocardiography (ECG), bleeding and clotting assessment, 3-dimensional (3D) vertebral reconstruction using CT, radiography of the relevant spinal region in two planes, and MRI. Heart and lung functions were also assessed in patients aged > 65 years. A standard clinical evaluation included medical history, physical examination of percussion pain, and assessment of pain intensity (visual analog scale [VAS]), activity level (Oswestry Disability Index [ODI]), and short-form 36 health survey domains of physical role (SF-36 rp) and bodily pain (SF-36 bp).

### Surgical technique

All surgeries were performed by the same senior chief physician under local anesthesia. Patients were placed in the prone position with the abdomen suspended. A 1-cm skin incision was made lateral to the desired percutaneous entry point at the pedicle. A trocar (Shandong Guanlong Medical Utensils Co. Ltd., Jinan City, Shandong Province, China) within a cannula was inserted into the pedicle as a working channel. After trocar removal, a balloon was inserted into the working channel and slowly inflated to create a low-pressure cavity for cement injection.

**Fig. 1:**
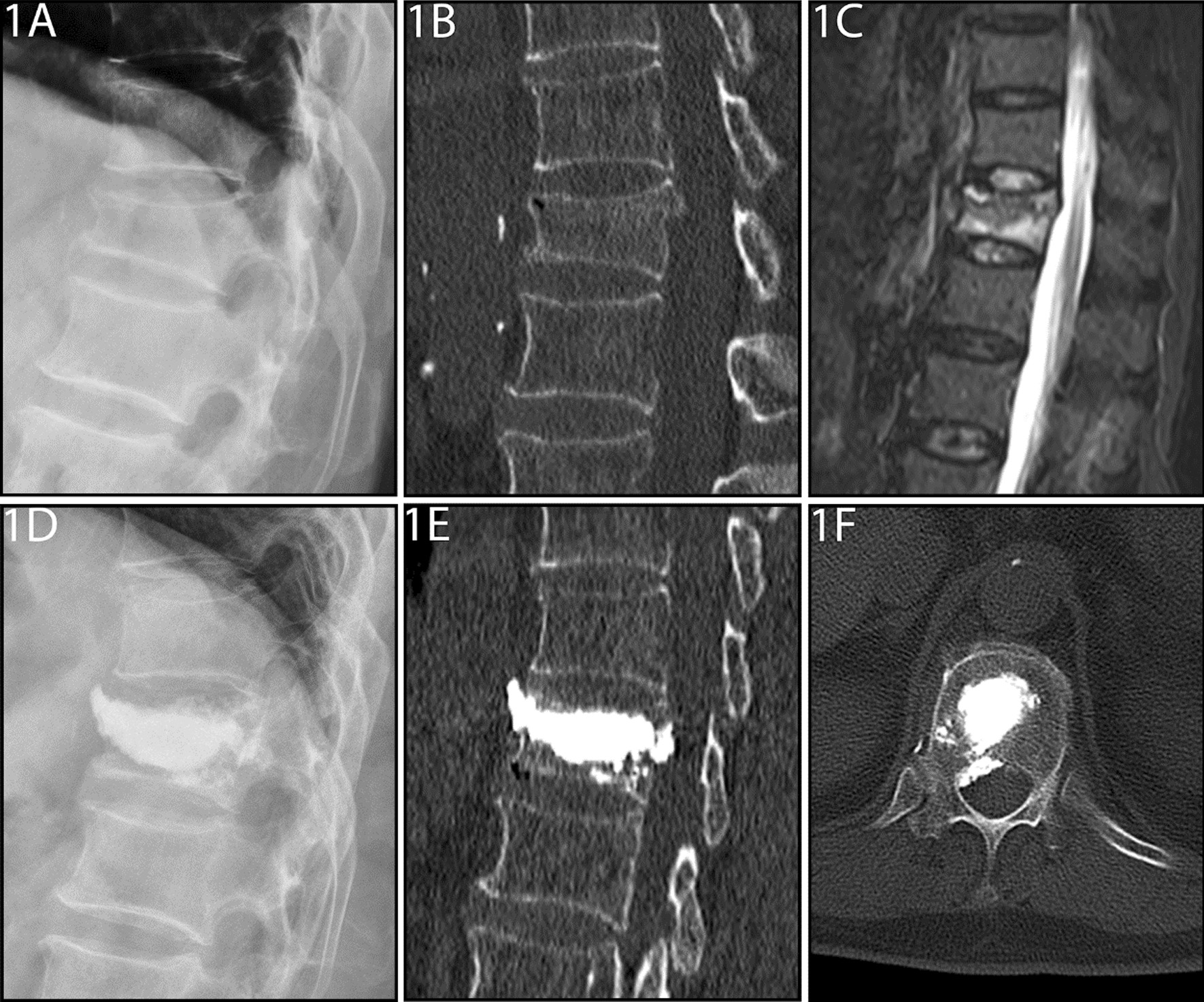
**A**–**F** A 77-year-old male patient with Kümmell’s disease at T12 in PKP group: **A** Lateral radiograph before surgery; **B** Intravertebral vacuum sign shown in sagittal computed tomography (CT) images before surgery; **C** A high signal intensity in the location of the cleft shown in sagittal T2-weighted MRI image before surgery; **D** Intervertebral cement leakage shown in lateral radiograph after surgery; **E**, **F** Intercranial cement leakage shown in sagittal and axial CT images after surgery

In the PKP group, poly-methyl methacrylate (PMMA) (Heraeus Medical, Germany) was injected into the diseased vertebra through the cannula under continuous fluoroscopic monitoring. PMMA injection was considered complete when it reached the posterior third of the vertebral body or until the point at which leaks through the cortical, epidural, and anterior veins were considered possible (Fig. 1).

**Fig. 2:**
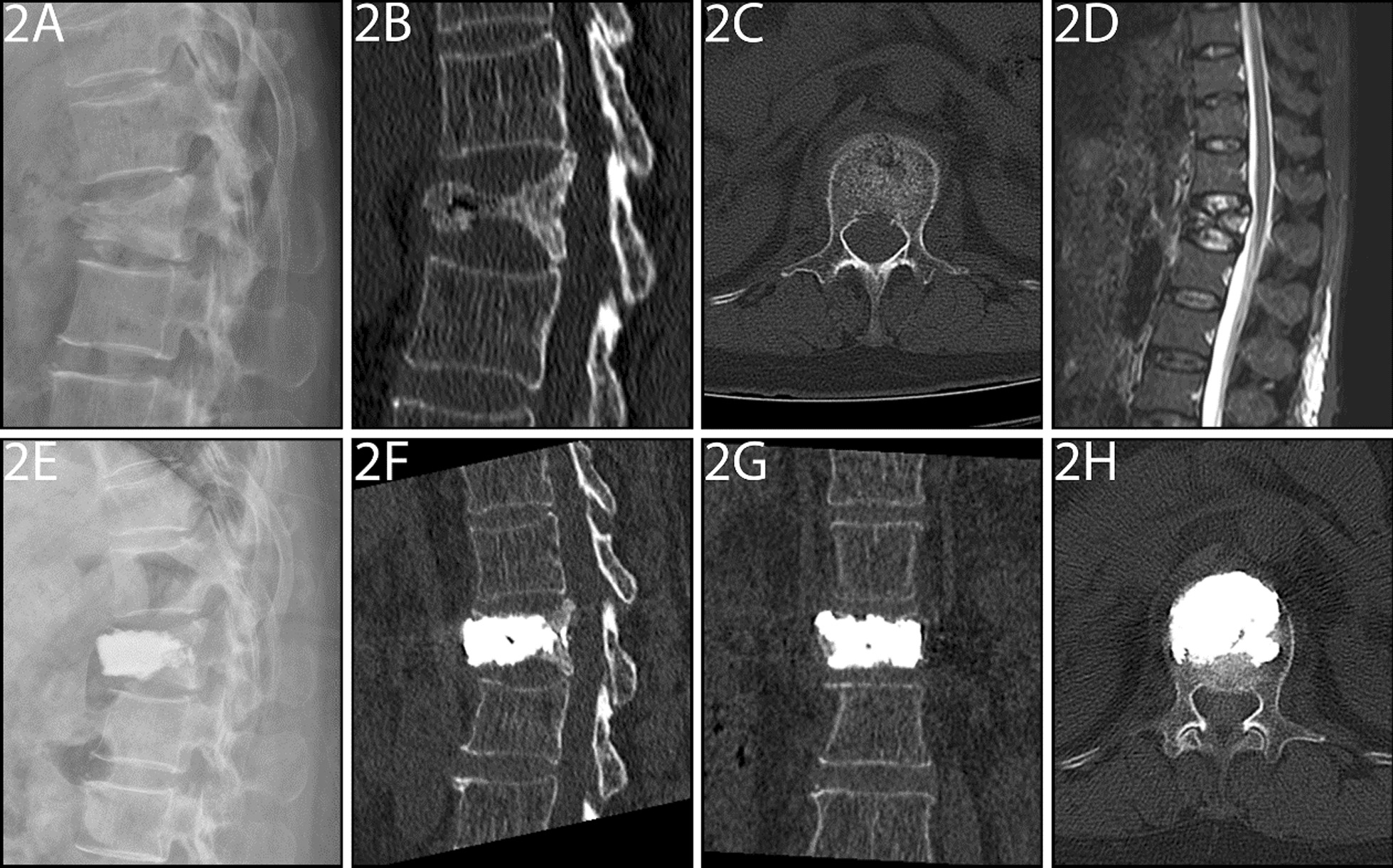
**A**–**H** A 83-year-old female patient with Kümmell’s disease at L1 in PMCP group: **A** Lateral radiograph before surgery; **B**, **C** Intravertebral vacuum sign shown in sagittal and axial computed tomography (CT) images before surgery; **D** A high signal intensity in the location of the cleft shown in sagittal T2-weighted MRI image before surgery; **E** Lateral radiograph after surgery; **F**–**H** Good cement distribution without leakage was shown in sagittal, coronal, and axial CT images after surgery

In the PMCP group, a mesh container (Shandong Guanlong Medical Utensils Co. Ltd.) was advanced into the cavity. Subsequently, PMMA cement was manually injected into the mesh container using a cement perfusion apparatus and under fluoroscopic guidance. Beyond a certain amount, the PMMA cement leaked outside the mesh container and entered the bone trabeculae (Fig. 2).

Patients underwent neurological examination immediately after surgery to assess for complications such as nerve damage. Postoperatively, the patients were encouraged to walk while wearing a 3-point fixation brace. Radiographs and CT images were obtained to evaluate the vertebral height, segmental kyphosis improvement, and cement distribution. Surgical time, cost, hospital stay, cement volume, and complications (cement leakage, cerebrospinal fluid leakage, and infection) were also noted. Back pain intensity was recorded using the VAS [15] and functional outcomes were evaluated using the ODI [16], SF-36 rp, and SF-36 bp [17]. All patients were followed up clinically and radiologically immediately, at 1, 3, and 6 months, and 1-year postoperatively.

Cobb’s angle and the anterior vertebral body height ratio (AVBHr) were measured on lateral radiographs [18, 19]. The cement distribution was calculated using CT images [20], and cement leakage was determined on X-ray and CT images.

Two, independent, blinded spine surgeons performed the clinical evaluations, while three assessed the radiographs.

### Statistical analysis

Statistical analyses were performed using the Statistical Package for the Social Sciences (version 19.0; IBM, Armonk, NY, USA). The numerical variables are presented as means ± standard deviations or medians (interquartile ranges). The Student’s t-test or Wilcoxon signed-rank test was used to compare measurements between the two groups. Repeated-measures analysis of variance was used to compare the VAS, ODI, SF-36, AVBHr, and Cobb’s angle preoperatively, immediately postoperatively, and 1-year postoperatively. Nominal variables (sex, cement leakage, segmental distribution, and trauma history) are presented as numbers (percentages) and compared using the Chi-square test. Statistical significance was set at a two-sided *p*-value < 0.05.

## Results

A total of 256 patients were divided into the PKP (122; 29 men, 93 women) and PMCP (134; 26 men, 108 women) groups according to the surgical treatment received. All patients were followed up for at least a year. The clinical characteristics of the patients are summarized in Table 1. There were no statistically significant differences in the demographic data, including age, sex, segmental distribution, T-score, body mass index, or trauma history, between the two groups. The mean cost in the PKP group was lower than that in the PMCP group (3697 ± 461 vs. 5255 ± 262 USD, *p* < 0.001). There were no significant differences in operative time, blood loss, cement volume, or hospital stay between the two groups (Table 2).

**Table 1:** Comparison of baseline data between two groups

	PKP (*n* = 122)	PMCP (*i* = 134)	*t* (*χ*^2^)	*p*
Age (years)	73.35 ± 8.28	75.49 ± 7.88	*t* = 0.123	0.902
Sex				
Male/female	29/93	26/108	*χ*^2^ = 0.722	0.395
BMI (kg/m^2^)	22.51 ± 3.61	23.01 ± 3.93	*t* = 1.059	0.291
BMD (T value)	− 3.03 ± 0.40	− 3.01 ± 0.32	*t* = − 0.376	0.708
Segments (cases)				
T10	4.00	8.00	*χ*2 = 1.396	0.845
T11	13.00	14.00		
T12	43.00	48.00		
L1	36.00	41.00		
L2	26.00	24.00		
Trauma history				
Yes (cases)	78	76	*χ*2 = 1.388	0.239
No (cases)	44	58		

*PKP* percutaneous kyphoplasty; *PMCP* percutaneous mesh-container-plasty

**Table 2:** Comparison of intraoperative conditions between two groups

	PKP (*n* = 122)	PMCP (*n* = 134)	*t* (*χ*^2^)	*p*
Operation time (min)	33.11 ± 3.65	33.69 ± 3.30	*t* = − 1.333	0.184
Hospital stays (days)	6.64 ± 3.02	7.20 ± 3.35	*t* = 1.406	0.161
Cost (dollar)	3697 ± 461	5255 ± 262	*t* = 33.600	< 0.001
Volume of cement(ml)	6.19 ± 1.27	6.42 ± 1.39	*t* = 1.399	0.163
Blood loss(ml)	5.82 ± 1.86	5.90 ± 1.92	*t* = 0.320	0.749
Cement leakage	35/122	23/134	*χ*2 = 4.840	0.028

*PKP* percutaneous kyphoplasty; *PMCP* percutaneous mesh-container-plasty

### Clinical evaluation

The immediate postoperative VAS scores and ODI values were significantly lower (*p* < 0.05), and the SF-36 rp and bp scores were significantly improved (*p* < 0.05) in both groups. There were no significant changes in the VAS, ODI, and SF-36 scores 1-year postoperatively. There were significant differences in the VAS and ODI scores between the two groups in the immediate postoperative period and 1-year postoperatively (*p* < 0.05); however, the difference in SF-36 scores was not statistically significant (*p* > 0.05) (Table 3).

**Table 3:** Clinical comparisons between the two groups

	PKP (*n* = 122)	PMCP (*n* = 134)	*t* (*Z*)	*p*
VAS				
Preoperative	6(6–7)	6(5–7)	*Z* = − 0.319	0.750
Postoperative	2(2–3)*	2(2–2)*	*Z* = − 3.123	0.002
1 year Postoperative	2(2–2)*	2(2–2)*	*Z* = − 2.630	0.009
ODI				
Preoperative	68.75 ± 6.64	67.70 ± 6.50	*t* = − 1.282	0.201
Postoperative	23.25 ± 3.50*	22.24 ± 3.55*	*t* = − 2.281	0.023
1 year Postoperative	19.64 ± 3.54*	18.58 ± 3.60*	*t* = − 2.363	0.019
SF-36 bp				
Preoperative	22(14.25–28.38)	21(15–22)	*Z* = − 1.059	0.290
Postoperative	84(84–84)*	84(84–84)*	*Z* = − 1.274	0.203
1 year Postoperative	84(84–84)*	84(84–84)*	*Z* = − 0.421	0.674
SF-36rp				
Preoperative	25(25–25)	25(25–25)	*Z* = − 0.008	0.993
Postoperative	75(75–75)*	75(75–75)*	*Z* = − 0.554	0.580
1 year Postoperative	75(75–75)*	75(75–100)*	*Z* = − 0.639	0.523

*Repeated measures variance analysis was used for the statistical analysis. There were significant differences (*p* < 0.05) between the postoperative or 1 year postoperative and preoperative values of these 2 groups

*PKP* percutaneous kyphoplasty; *PMCP* percutaneous mesh-container-plasty; *VAS* visual pain analog scale; *ODI* Oswestry disability index; *SF-36 rp* short-form 36 health survey domains role physical; *SF-36 bp* short-form 36 health survey domains bodily pain

Therefore, PMCP had advantages over PKP in terms of pain relief and functional recovery for the treatment of Kümmell's disease.

### Radiologic evaluation

The AVBHr and Cobb’s angle improved in the PKP group (preoperative: 70.85 ± 16.62% and 17.29 ± 9.78°; postoperative: 80.28 ± 13.02% and 13.05 ± 8.40°, respectively) and PMCP group (preoperative: 70.96 ± 18.01% and 17.01 ± 10.53°; postoperative: 84.81 ± 12.96% and 10.76 ± 9.23°, respectively) (*p* < 0.05). However, 1-year follow-up showed no significant changes in the AVBHr or Cobb's angle in the PKP and PMCP groups. In both groups, AVBHr and Cobb angles significantly improved. The PMCP group had significantly higher cement distribution than the PKP group had (41.81 ± 8.82% vs. 33.65 ± 9.24%, *p* < 0.001). The radiographic results are presented in Table 4. PKP and PMCP restored the height and improved segmental kyphosis of the vertebrae significantly. PMCP improved cement distribution, vertebral height, and segmental kyphosis better than PKP did in the treatment of Kümmell's disease.

**Table 4:** Radiologic comparisons between the two groups

	PKP (*n* = 122)	PMCP (*n* = 134)	*t*	*p*
AVBHr (%)				
Preoperative	70.85 ± 16.62	70.96 ± 18.01	0.054	0.957
Postoperative	80.28 ± 13.02*	84.81 ± 12.96*	2.791	0.006
1 year Postoperative	75.44 ± 15.05*	81.69 ± 13.79*	3.467	0.001
The Cobb angle (°)				
Preoperative	17.29 ± 9.78	17.01 ± 10.53	− 0.221	0.826
Postoperative	13.05 ± 8.40*	10.76 ± 9.23*	− 2.071	0.039
1 year Postoperative	15.39 ± 9.01*	12.86 ± 9.68*	− 2.163	0.031
Cement distribution (%)	33.65 ± 9.24	41.81 ± 8.82	7.234	< 0.001

*Repeated measures variance analysis was used for the statistical analysis. There were significant differences (*p* < 0.05) between postoperative or 1 year postoperative and preoperative values of these 2 groups

*PKP* percutaneous kyphoplasty; *PMCP* percutaneous mesh-container-plasty; *AVBHr* anterior vertebral body height ratio

### Surgical complications

**Fig. 3:**
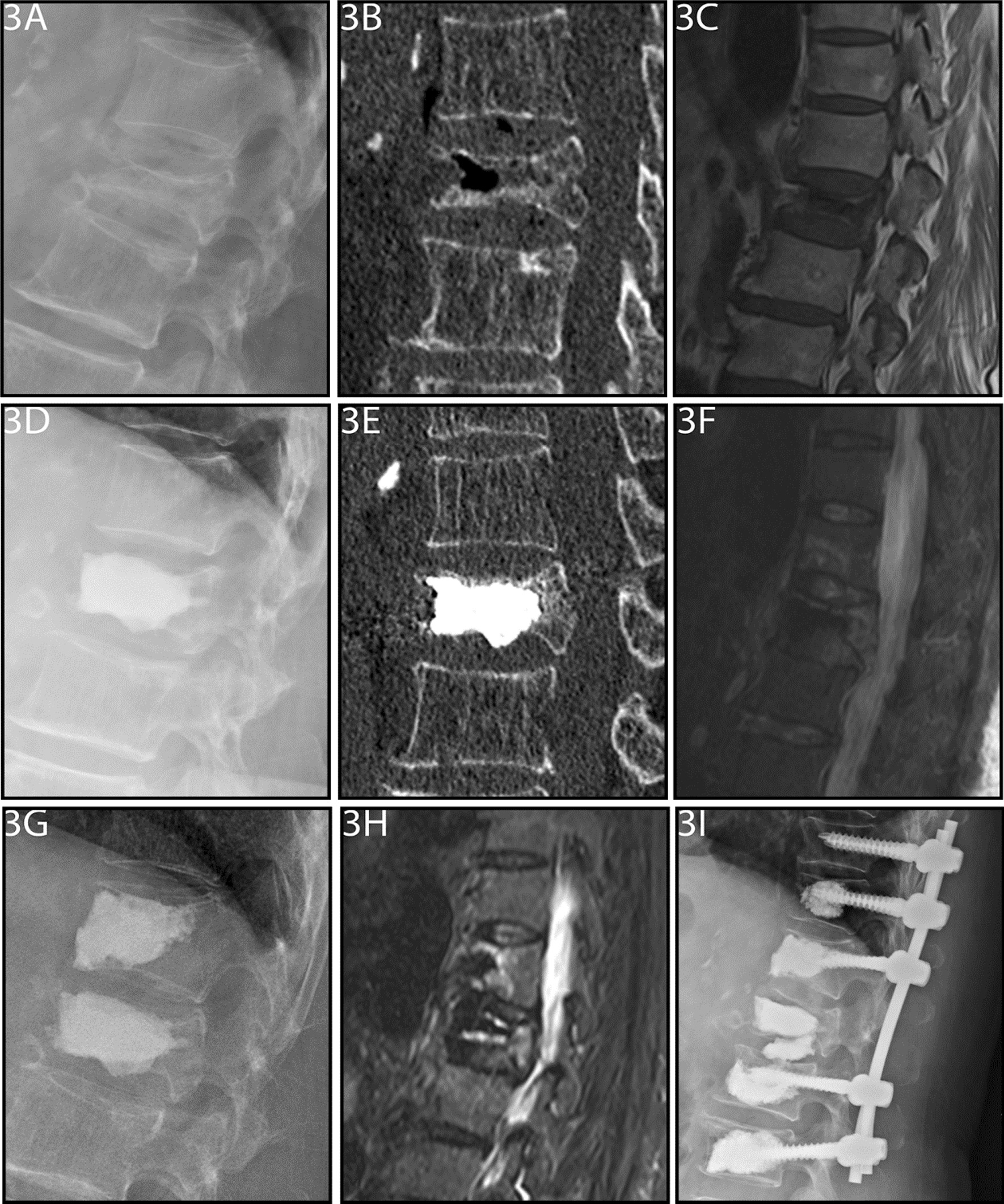
**A**–**I** A 75-year-old female patient with Kümmell’s disease at L1 in PKP group: **A** Lateral radiograph before surgery; **B** Intravertebral vacuum sign shown in sagittal computed tomography (CT) images before surgery; **C** A low signal intensity in the location of the cleft shown in sagittal T1-weighted MRI image before surgery; **D**–**E** Intervertebral cement leakage is shown in lateral radiograph and sagittal CT images after surgery; **F** An adjacent fracture was noted in MRI image one month after PKP surgery. **G** Lateral radiograph after secondary PKP surgery; **H** MRI image showed the fracture remained nonunion about three months after secondary PKP surgery; **I** Lateral radiograph after internal fixation with cement augmentation

Immediate postoperative CT revealed cement leakage in 29% (35/122) of patients in the PKP group (10 in the intervertebral space, 23 in the paravertebral tissues or veins, and two in the canal) and in 17% (23/134) of patients in the PMCP group (eight in the intervertebral space, 15 in the paravertebral tissues or veins, and none in the canal) (*p* < 0.05) (Table 2). The two patients with cement leakage into the canal presented with lower-extremity radiating pain, numbness, and decreased muscle strength postoperatively. After treatment to reduce nerve root edema and improve its nutrition, the symptoms gradually relieved in one patient. Surgical intervention was required in the other patient because the symptoms persisted. During postoperative rehabilitation, one patient each in the PKP (0.8%) and PMCP (0.7%) groups developed an infectious fever (Table 5). After supportive treatment, the patient’s body temperature returned to normal. Acute pulmonary embolism was not observed in either group. During the follow-up period, an adjacent vertebral fracture was observed in nine (7.4%) and seven (5.2%) patients in the in the PKP and PMCP groups, respectively (Table 5); however, the difference was not statistically significant. One patient in the PKP group developed an adjacent vertebral fracture one month after surgery, which was managed with another PKP. However, three months after surgery, fracture non-union was seen. Hence, internal fixation with cement augmentation was performed (Fig. 3).

**Table 5:** Incidence of complications in the two groups

Complication	PKP (*n* = 122)	PMCP (*n* = 134)
Acute pulmonary embolism	0	0
Nerve damage	2	0
Infectious fever	1	1
Cement leakage	35	23
Adjacent vertebral fracture	9	7

*PKP* percutaneous kyphoplasty; *PMCP* percutaneous mesh-container-plasty

## Discussion

Ineffective treatment of osteoporotic vertebral compression fractures can lead to non-union and delayed vertebral collapse, resulting in Kümmell's disease [21], whose main pathogenesis includes ischemic osteonecrosis and pseudoarthrosis [7, 22, 23]. PKP has been widely used in patients with osteoporotic Kümmell's disease and neurological integrity, especially in those intolerant to general anesthesia [24–26], with good results [27].

PKP is associated with postoperative complications such as the risk of cement leakage. In order to reduce the occurrence of cement leakage, mesh containers have been used in PVP [28]. Currently, both PKP and PMCP are important procedures for the treatment of Kümmell's disease.

In our study, the VAS and ODI scores at different postoperative time points significantly improved in the two groups, compared to those on the day before surgery; the differences were statistically significant. These results suggest that PMCP has advantages over PKP in terms of pain relief and functional recovery for the treatment of Kümmell's disease. Furthermore, PMCP is safer than PKP, with better cement distribution, vertebral height restoration, and improvement of segmental kyphosis.

Researchers have observed that the low-density shadow in the fissure sign of Kümmell's disease continues to the outside of the vertebral body wall, which is larger. Cement may enter the paravertebral tissue through the anterior fissure and burn the adjacent blood vessels. Alternatively, it may enter the spinal canal through the posterior wall fissure, causing damage to the spinal cord or nerve roots. Therefore, Kümmell's disease has a higher risk of cement leakage than general osteoporotic vertebral fractures alone [29, 30]. The mesh container used in this study was a newly developed inflatable mesh bone filler designed by Shandong Guanlong Medical Products Co., Ltd., Shandong, China. It has a dense mesh structure composed of polyethylene terephthalate fibers. In the PMCP technique, a cavity is formed in the treated vertebral body by applying a bone-expansion brace. Following the removal of the brace, a mesh container is inserted into the cavity. After insertion, the mesh container is filled with PMMA cement. As the mesh container expands, it reaches the cavity edge. The mesh container exerts pressure on the surrounding bone tissues, gradually restoring the vertebral body height. When the perfusion pressure reaches a certain degree, bone cement leaks outside of the mesh container and enters the bone trabeculae, strengthening and stabilizing it.

The amount and timing of cement injection reportedly affects the leakage rate. The injection volume for vertebroplasty should be 16–30% of the vertebral body volume, which is 4–6 ml [31]. Ryu et al. found that a larger cement volume could lead to a higher incidence of epidural cement leakage [32]. Fu et al. determined a positive dose–response correlation between the cement volume and incidence of cement leakage [33]. In this study, there was no significant difference in the cement volume between the two groups, which supports our findings. The timing of the cement injection was also an important factor; early injections resulted in better cement distribution and a higher probability of leakage. However, late injections resulted in relatively poor cement distribution and less leakage. In the present study, all procedures were performed by the same senior surgeon and the injection timing was defined as the time the cement was drawn. These minimized the potential influence of the cement injection timing on the leakage rate. In our study, cement leakage was determined in 29% (35/122) and 17% (23/134) of patients in the PKP and PMCP groups, respectively. Therefore, PMCP has a better ability to inhibit cement leakage than PKP does for the treatment of Kümmell's disease.

A greater bone cement distribution reportedly indicates a greater anterior vertebral height restoration and Cobb’s angle correction [34]. When the cement volume remains constant, extensive cement distribution can effectively improve the kyphotic angle and vertebral height without causing cement leakage or adjacent vertebral fractures [35]. Adequate contact between the cement and upper and lower endplates can restore vertebral strength, maintain vertebral height, and reduce the risk of vertebral recompression and long-term pain [36]. In the present study, the cement distribution rate was higher in the PMCP group than in the PKP group (41.81 ± 8.82 vs, 33.65 ± 9.24). Height restoration and improvement in segmental kyphosis were both greater in the PMCP group than in the PKP group. A possible mechanism for the correlation between height restoration and kyphosis was the inflation of the mesh container. In the PKP technique cement is injected after the balloon has been expanded and withdrawn. In such cases, the vertebral body is prone to "rebound" and may cause height loss again. The mesh container can effectively compensate for this deficiency by slowly dispersing the cement through the mesh holes, and effectively control cement leakage through the combination of cement and bone tissue strands. Thus, the vertebral height recovery and cement injection is synchronized, with no "rebound" phenomenon.

Adjacent vertebral fractures occurred in nine and seven patients in the PKP and PMCP groups. No statistically significant differences were found between the two groups. Fracture of the adjacent vertebral body may be caused by the high strength of PMMA cement, causing degeneration of the adjacent intervertebral disc and reducing its cushioning effect [37].

The present study had several limitations. This was a retrospective study with an inherent bias. The sample size was obtained from a single center. Prospective randomized controlled studies with a larger sample size and long-term follow-up are needed to evaluate the clinical and radiographic efficiency of PMCP more reliably and objectively.

## Conclusion

There is significant evidence that PMCP has advantages over PKP in terms of pain relief and functional recovery for the treatment of Kümmell's disease. Moreover, PMCP is more effective than PKP in preventing cement leakage, increasing cement distribution, and improving vertebral height and segmental kyphosis, despite its higher cost.

## Data Availability

The patients’ data were collected in the Third affiliated Hospital of Wenzhou Medical University.

## Abbreviations

PKP: Percutaneous kyphoplasty
PMCP: Percutaneous mesh-container-plasty
PMMA: Poly-(methyl methacrylate)
ODI: Oswestry disability index
VAS: Visual analogue scale
AVBHr: Anterior vertebral body height ratio
